# Sensitivity of African swine fever virus to type I interferon is linked to genes within multigene families 360 and 505

**DOI:** 10.1016/j.virol.2016.03.019

**Published:** 2016-06

**Authors:** Josephine P. Golding, Lynnette Goatley, Steve Goodbourn, Linda K. Dixon, Geraldine Taylor, Christopher L. Netherton

**Affiliations:** a Vaccinology Group, The Pirbright Institute, Ash Road, Pirbright, Surrey GU24 0NF, UK; b ASFV Group, The Pirbright Institute, Ash Road, Pirbright, Surrey GU24 0NF, UK; c Institute for Infection and Immunity, St. George’s, University of London, London SW17 0RE, UK

**Keywords:** African swine fever virus, Interferon, Multigene family, Pig, Cytokine, Innate immunity, Immune evasion, Asfarviridae, Macrophage, Dendritic cell

## Abstract

African swine fever virus (ASFV) causes a lethal haemorrhagic disease of pigs. There are conflicting reports on the role of interferon in ASFV infection. We therefore analysed the interaction of ASFV with porcine interferon, *in vivo* and *in vitro*. Virulent ASFV induced biologically active IFN in the circulation of pigs from day 3-post infection, whereas low virulent OUR T88/3, which lacks genes from multigene family (MGF) 360 and MGF505, did not. Infection of porcine leucocytes enriched for dendritic cells, with ASFV, *in vitro*, induced high levels of interferon, suggesting a potential source of interferon in animals undergoing acute ASF. Replication of OUR T88/3, but not virulent viruses, was reduced in interferon pretreated macrophages and a recombinant virus lacking similar genes to those absent in OUR T88/3 was also inhibited. These findings suggest that as well as inhibiting the induction of interferon, MGF360 and MGF505 genes also enable ASFV to overcome the antiviral state.

## Highlights

• Virulent strains of African swine fever virus induces interferon during infection.
• Virulent, but not attenuated strains of ASFV are resistant to the antiviral effects of interferon in porcine macrophages.
• Sensitivity to interferon is linked to genes within multigene family 360 and multigene family 530.
• Dendritic cells are a potential source of the interferon detected *in vivo*.

## 1 Introduction

African swine fever (ASF) is a viral haemorrhagic disease of domestic and wild pigs (both *Sus scrofa*). Virulent strains of ASF virus (ASFV) can cause up to 100% mortality, while low virulent strains can cause inapparent infections from which the animals develop immunity to related virulent strains. The natural hosts for the virus are warthogs (*Phacochoerus aethiopicus*) and bushpigs (*Potamochoerus porcus*), which do not develop clinical signs of disease (Anderson et al., 1998, Oura et al., 1998, Thomson, 1985, Thomson et al., 1980). In addition, ASFV can infect soft ticks of the family *Ornithodoros* (Boinas et al., 2011, Diaz et al., 2012), which can contribute to transmission of the virus in the field. Controlling outbreaks of ASFV can prove to be difficult, as evidenced by the continuing problems in the Russian Federation, and surrounding European countries. Understanding the immune response to ASFV is of importance to delivering an effective vaccine against the virus.

ASFV is the only member of the *Asfarviridae* family, and is grouped within the nucleo-cytoplasmic large DNA viruses. ASFV is a non-segmented, linear, double-stranded DNA virus with a genome length ranging from 170 to 193 kilo base pairs which codes for between 151 and 167 open reading frames (Chapman et al., 2008, de Villiers et al., 2010, Dixon et al., 2013, Yáñez et al., 1995). Length variations in the genome occur at the termini, due to insertions and deletions of genes within five multigene families (MGF). The function of the genes within these families is currently unknown, and they do not share sequence identity with other known genes. However, previous work suggests that genes within MGF360 and MGF505 (also referred to as MGF530) are important in determining host range (Burrage et al., 2004, Zsak et al., 2001) and are involved in inhibition of interferon (IFN) induction (Afonso et al., 2004).

Type I IFN is a crucial component of the innate response to viral infection (Randall and Goodbourn, 2008). Components of pathogens such as nucleic acids are recognised by intracellular and extracellular receptors that induce a complex signal transduction pathway that leads to the secretion of type I IFN; these cytokines in turn induce the differential expression of hundreds of different genes (Der et al., 1998), leading to the establishment of an antiviral state within neighbouring cells and subsequent restriction of further rounds of viral replication. IFNβ is induced in most cell types, whereas IFNα shows a more restricted pattern of induction; notably, plasmacytoid dendritic cells (pDCs), also called natural interferon producing cells, are capable of producing large amounts of IFNα due to constitutive expression of the transcription factor IRF7. In addition to directly restricting viral replication, IFNα/β up-regulates surface expression of MHC class I and II, activates dendritic cells (DCs) and natural killer cells (Carrero, 2013, Nguyen et al., 2002), and can influence T-cell function (Huber and Farrar, 2011). As such, IFN has an important role to play in both innate and adaptive immunity to pathogens.

ASFV primarily replicates in monocytes and macrophages, although other cells can be infected at later time points following infection of pigs (Pérez et al., 1994, Ramiro-Ibáñez et al., 1995). Avirulent/low-virulent strains, that lack genes from MGF360 and MGF505, induce the expression of IFN during infection of macrophages *in vitro* (Afonso et al., 2004, Gil et al., 2008), whereas virulent ASFV strains do not (Afonso et al., 2004, Gil et al., 2008, Zhang et al., 2006). However, IFNα and IFNβ have been detected in the serum of pigs infected with virulent Georgia 2007/1 (Karalyan et al., 2012), demonstrating that while virulent ASFV does not induce IFN *in vitro*, it does induce it *in vivo*.

There are conflicting reports on the role of IFN during ASFV infection. Replication of two virulent ASFV strains was reduced in porcine macrophages pretreated with bovine IFNα (Esparza et al., 1988). A similar observation was seen in Vero cells pretreated with human IFNα and then infected with cell culture-adapted ASFV BA71V (Paez et al., 1990). In contrast, induction of IFN in porcine macrophages by polyI:C did not affect the replication of either the virulent Kirawira or the attenuated Uganda strain of ASFV (Wardley et al., 1979). These apparent discrepancies in the sensitivity of ASFV to IFN, coupled with the differences seen between the induction of IFN *in vitro* and *in vivo*, led us to re-examine this important host-pathogen interaction. We compared the induction of IFN in pigs infected with virulent or low virulent ASFV strains and the effect of porcine IFNα on ASFV replication *in vitro*.

## 2 Results

### 2.1 Characterisation of the systemic IFN response to infection of pigs with virulent and low virulent ASFV

**Fig. 1:**
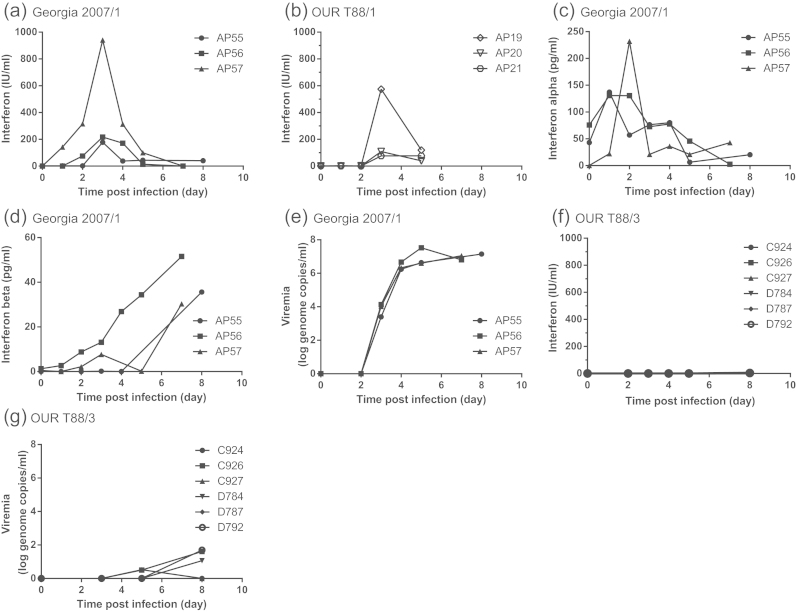
IFN in the serum of pigs infected with ASFV Georgia 2007/1, OUR T88/1 or OUR T88/3 and the level of viraemia estimated by qPCR. Serum was collected from outbred Large White-Landrace pigs inoculated intramuscularly with 10^4^ HAD50 of virulent Georgia 2007/1 (a, c–e) or virulent OUR T88/1 (b). Biologically active IFN was determined using the MxA-CAT assay (a and b). Results were expressed as the mean of IFN IU/ml of a single biological sample which was measured in duplicate on the CAT ELISA. IFNα was determined using a porcine IFNα ELISA (c). Results were expressed as the mean of IFNα pg/ml of duplicate samples. IFNβ was determined using a porcine IFNβ ELISA. (d). Results were expressed as the mean of IFNβ pg/ml of duplicate samples. Serum was collected from NIH minipigs inoculated intramuscularly with 10^4^ TCID50 of low virulent OUR T88/3 (f and g). Biologically active IFN was determined using the MxA-CAT assay (f). Viraemia was estimated by qPCR and expressed as ASFV genome copy per ml blood (log_10_) in Large White Landrace pigs inoculated with virulent Georgia 2007/1 (e) or NIH minipigs inoculated with attenuated OUR T88/3 (g). Numbers AP19, AP55, C924 *etc.* correspond to animal numbers.

Serum levels of IFN were determined in pigs infected either with the virulent Georgia 2007/1, or the low virulent OUR T88/3 strains of ASFV using a bioassay that measures CAT expression controlled by the promoter of the IFN-stimulated gene MxA (Fray et al., 2001), and by IFNα- and IFNβ-specific ELISAs. Following infection with Georgia 2007/1, the highest levels of IFN activity were observed on days 3 to 4 post-infection (p.i.) (Fig. 1a). The quantity of biologically active IFN measured at these time points varied considerably between pigs, from approximately 200 IU/ml in pig AP55 to 1000 IU/ml in pig AP57. Sera from three pigs infected with virulent OUR T88/1 (Abrams et al., 2013) contained similar levels of biologically active IFN as the animals infected with Georgia 2007/1, ranging from 100 IU/ml to nearly 600 IU/ml between day 3 and 5 p.i. (Fig. 1b). Pigs infected with virulent Georgia 2007/1 produced IFNα and IFNβ profiles similar to those seen in previous studies (Karalyan et al., 2012). IFNα peaked at day 1 or 2 p.i., measuring between 130 and 230 pg/ml (Fig. 1c), and then decreased until the end of the study. IFNβ was detected in the serum later during infection than IFNα and the trend was for a general increase in IFNβ as disease progressed. The highest amounts of IFNβ detected were on the final day (day 7–8 p.i.) of the experiment and ranged from 30 to 52 pg/ml (Fig. 1d). Viraemia was first detected on day 3 p.i. in pigs infected with Georgia 2007/1 and increased until day 5 when virus levels plateaued between 10^6^ and 10^7^ genome copies per ml (Fig. 1e). The appearance of circulating IFN preceded the appearance of virus by approximately one to two days. These data show that infection with virulent ASFV induces IFN in the circulation, which, in some individuals, reaches the equivalent of nearly 1000 IU/ml (Fig. 1a). To analyse the IFN response following infection with low virulent ASFV, six NIH minipigs and five outbred pigs were infected with the OUR T88/3 strain. Biologically active IFN was not detected in the serum of any of the NIH minipigs (Fig. 1f) or outbred pigs (Supplemental Fig. 1) infected with OUR T88/3. In contrast to pigs infected with Georgia 2007/1, only very low levels (<10^2^ genome copies/ml) of circulating virus were detected in four of the OUR T88/3-infected minipigs (Fig. 1g). These data show a clear difference *in vivo* in the induction of IFN in pigs infected with virulent and low virulent ASFV. Higher levels of biologically active IFN in pigs infected with Georgia 2007/1 appeared to coincide with the appearance of viraemia at day 3 (Fig. 1e). Importantly viraemia increased between day 3 and 5 despite the presence of a significant amount of biologically active IFN.

### 2.2 Replication of virulent ASFV is not inhibited by IFNα

**Fig. 2:**
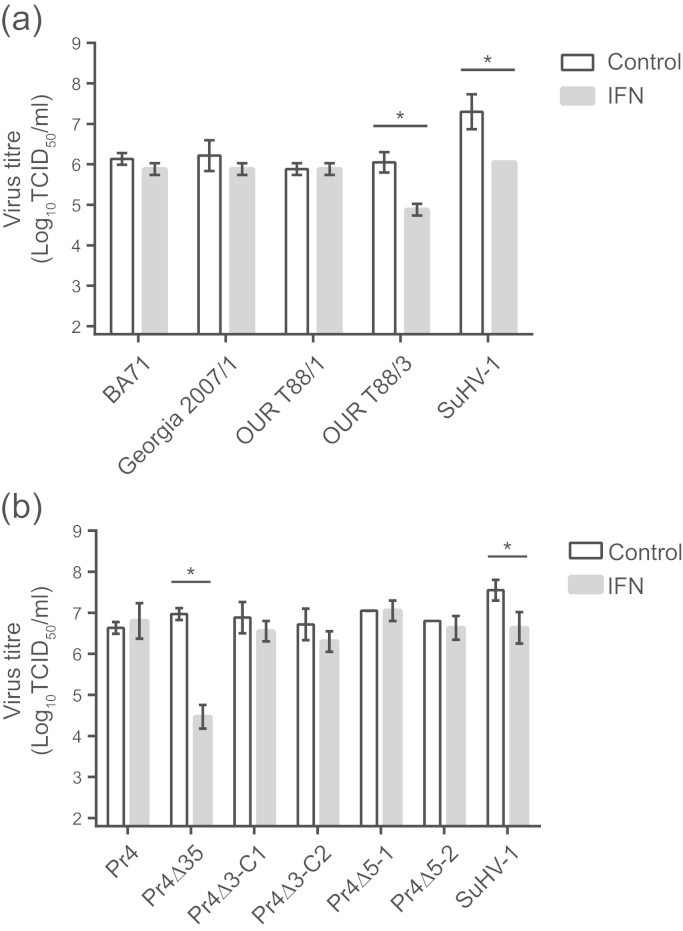
Replication of ASFV strains lacking genes from MGF360 and MGF505 in the presence of porcine IFNα. Alveolar macrophages were infected with the indicated field ASFV strains (a) and/or recombinant ASFV strains (b) as well as suid herpesvirus 1 (SuHV-1) at an MOI of 0.1. Cells were pretreated for 24 h prior to infection with 2000 IU/ml recombinant porcine IFNα (grey bars) or left untreated (white bars). Virus yields were determined 48 h post infection on alveolar macrophages. Titres are shown as the mean log_10_TCID_50_/ml±standard deviation of triplicate wells and are representative of three independent experiments.

The finding that virulent ASFV continues to replicate in the presence of circulating IFN does not correlate with previously published *in vitro* data demonstrating that virulent ASFV is sensitive to the antiviral effects of IFNα (Esparza et al., 1988). In order to investigate the antiviral effects of IFN on ASFV further, porcine alveolar macrophages, pretreated for 24 h with recombinant porcine IFNα, were infected with virulent ASFV isolates BA71, Georgia 2007/1 and OUR T88/1, the low virulent OUR T88/3 isolate, as well as suid herpesvirus 1 (SuHV-1 also referred to as pseudorabies and Aujesky׳s disease virus) which is sensitive to porcine IFNα (Cheng et al., 2006). IFNα did not affect the ability of any of the virulent ASFV strains tested to replicate in alveolar macrophages. However, the replication of both OUR T88/3 and SuHV-1 were reduced approximately 10-fold by pre-treatment of cells with 2000 IU/ml of recombinant IFNα (Fig. 2a). Similar results were also observed in macrophage cultures derived from blood monocytes (Supplemental Fig. 2). The observation that Georgia 2007/1 and OUR T88/1 were not inhibited by IFNα *in vitro* is consistent with our results showing that these isolates replicate *in vivo* in the presence of IFN (Fig. 1), and contrasts with a previous report showing that replication of virulent BA71 in porcine macrophages was inhibited by recombinant bovine IFNα (Esparza et al., 1988).

### 2.3 Role of MGF360 and MGF505 genes in overcoming the antiviral effects of IFN

In comparison with virulent ASFV, OUR T88/3 lacks five genes from MGF360 (MGF360-10L, -11L, -12L, -13L, and -14L) and two genes from MGF505 (MGF505-1R and -2R) (Chapman et al., 2008). Recombinant viruses derived from the virulent Pr4 isolate of ASFV in which genes from MGF360 and MGF505 were deleted (Burrage et al., 2004) were used to further refine the role of these gene families in overcoming the antiviral effects of IFN. Pr4Δ35 is a deletion mutant that lacks the genes that are also absent in OUR T88/3, as well as an additional MGF360 gene (MGF360-9L). Pr4Δ3-C1 lacks MGF360-9L, -10L, and -11L; Pr4Δ3-C2 lacks MGF360-12L, -13L, and -14L; Pr4Δ5-1 lacks MGF505-1R; and Pr4Δ5-2 lacks MGF505-2R. Previous studies have shown that Pr4Δ35 induces IFNα in porcine macrophages, *in vitro*, whereas the parental Pr4 strain does not (Afonso et al., 2004). Replication of Pr4Δ35 was significantly reduced in alveolar macrophages treated with 2000 IU/ml of recombinant IFNα. In contrast, the replication of the parental Pr4 virus, as well as all of the other recombinants, was not inhibited by IFNα (Fig. 2b). These observations suggest that no single gene is sufficient to enable ASFV to replicate optimally in the presence of IFNα.

### 2.4 Potential source of IFN in animals infected with virulent ASFV

**Fig. 3:**
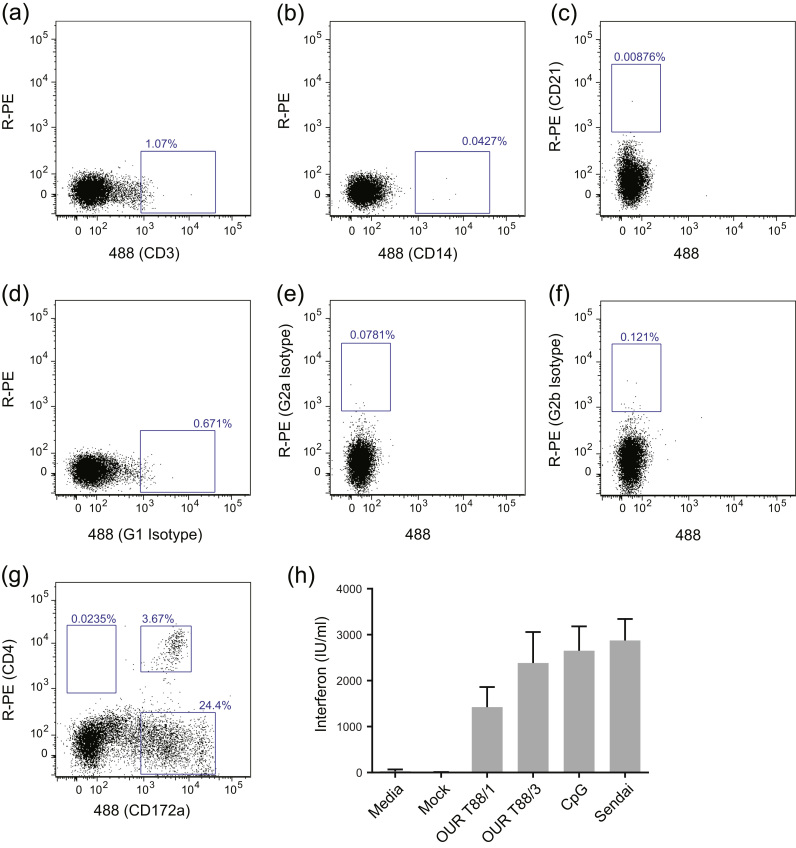
Virulent ASFV induces IFN in CD3-CD14-CD21-depleted peripheral blood mononuclear cells (PBMCs). PBMCs were purified from the blood of three animals and then depleted of CD3^+^, CD14^+^ and CD21^+^ cells using monoclonal antibodies and MACS beads. Depleted cells were stained with antibodies against CD3, CD14, CD21, CD4, CD172a as well as with appropriate isotype controls and analysed by flow cytometry, representative results from one pig are shown. The blue boxes show populations of interest and the percentage of the total, live cells are indicated (a–g). Depleted cells derived from the three pigs were incubated for 24 h with media alone; OUR T88/1, OUR T88/3, or mock (MOI 1); or with CpG or Sendai virus as positive controls. Biologically active IFN was determined from duplicate biological replicates using the MxCAT assay (h) and the results are expressed as the mean of IFN IU/ml of the three pigs±standard deviation.

Our data show that virulent ASFV is able to induce large amounts of circulating IFN in infected animals. Previous data shows that virulent ASFV does not induce IFN in porcine macrophage cultures and similarly we could only detect biologically active IFN in the supernatants of macrophages infected with OUR T88/3 or Pr4Δ35 (Supplemental Fig. 3) Therefore we looked to see whether IFN is produced by ASFV-infected DCs. Peripheral blood mononuclear cells (PBMCs) from naïve pigs were depleted of T-cells, B-cells and monocytes using a cocktail of monoclonal antibodies in order to enrich for DCs. These negatively-selected cells were then incubated with virulent OUR T88/1, low virulent OUR T88/3, CpG or Sendai virus for 24 h. Supernatants from these cultures were then analysed for IFN activity. An example of the proportion of PBMCs expressing CD3, CD14 or CD21 following depletion is shown in Fig. 3a–c. The negatively-selected cell population comprised ~20% CD172a^+^ cells consisting of both CD172a high and CD172a low cells. A proportion of the CD172a low population also expressed CD4 which is characteristic of pDCs (Fig. 3g) (Calzada-Nova et al., 2010, Summerfield et al., 2003). This negatively-selected cell population produced large quantities of biologically active IFN when exposed to both virulent and low virulent strains of ASFV, as well as to CpG and Sendai virus (Fig. 3h). IFN was not detected in supernatants from cells incubated in media alone, mock-infected cells (Fig. 3h) or ASFV isolates incubated for 24 h in the absence of any cells (not shown). These observations suggest that pDCs may contribute to the high levels of IFN detected in animals infected with virulent strains of ASFV.

## 3 Discussion

IFN is an important component of the innate response to infection and many viruses have evolved mechanisms to avoid or abrogate IFN induction and/or the antiviral state. Previous work suggested that ASFV was sensitive to the effects of IFN (Esparza et al., 1988, Paez et al., 1990) and this correlated with the apparent link between attenuation and the ability to induce IFN *in vitro* (Afonso et al., 2004, Gil et al., 2008, Powell et al., 1996, Zhang et al., 2006). However, recent experiments revealed the presence of IFN in the serum of animals infected with virulent viruses shedding doubt on this hypothesis (Karalyan et al., 2012). In this study, we confirm that IFNα and IFNβ are present in the serum of animals infected with virulent ASFV (Fig. 1c and d) and also demonstrate the presence of biologically active IFN in the circulation (Fig. 1a and b). The appearance of IFN in the blood is co-incident with the appearance of viraemia (Fig. 1e) and appears to correlate with the appearance of TNFα (Gómez del Moral et al., 1999) and IL-1β (Salguero et al., 2002). The profile of IFN activity in the serum of animals infected with virulent ASFV was very similar to that observed in pigs infected with virulent classical swine fever virus (CSFV), although IFNβ was not detected in these pigs (Summerfield et al., 2006). The presence of high levels of IFN has been linked to the depletion of B-and T-cells from the blood of CSFV-infected pigs and IFN, in combination with TNFα, could contribute to the lymphopenia observed during acute ASF (Sanchez-Vizcaino et al., 1981).

The large amount of biologically active IFN in the circulation, several days before viraemia reached its peak, suggested that IFN did not inhibit virus replication *in vivo*. This was inconsistent with previous studies showing that replication of ASFV was sensitive to IFN *in vitro* (Esparza et al., 1988). Our studies did not confirm this observation and in fact we demonstrated that replication of virulent BA71 was not inhibited in either alveolar (Fig. 2a) or blood-derived macrophages (Supplemental Fig. 2) treated with porcine IFNα. In our hands, the replication of a number of different virulent ASFV isolates from different genetic backgrounds was not inhibited by IFN in both macrophage models. Therefore, our results with virulent ASFV are in agreement with those of Wardley et al. (1979).

One apparent contradiction in the literature explored here was the presence of IFN in the serum of pigs with acute ASF (Karalyan et al., 2012) and the failure of virulent viruses to induce IFN in macrophages *in vitro* (Afonso et al., 2004, Gil et al., 2008, Zhang et al., 2006). This may be simply due to significant differences between the types of macrophages that ASFV infects *in vivo* and the tractable porcine macrophage models available *in vitro*. Another possibility is that IFN is being secreted by neighbouring cells in response to signals such as cGAMP(2′-5′) emanating from ASFV-infected cells (Ablasser et al., 2013). Alternatively IFN may be secreted by cells such as DCs in response to ASFV infection. Many types of DCs can secrete IFN in response to viral infection (O’Keeffe et al., 2012, Schulte et al., 2015) and pDCs in particular secrete large quantities of IFN in response to viral infection. A population of PBMCs enriched for CD4^+^/CD172^+^ cells produced large quantities of IFN when exposed to both virulent and low virulent ASFV. This is the first example of a cell population secreting IFN in response to virulent ASFV infection and implicates pDCs as a potential source of the IFN found in the serum of animals undergoing an acute ASF infection. Future experiments should attempt to define the types of IFN secreted by these DCs and to further characterise the DC population responsible *in vitro* and by defining IFN secreting cells *in vivo*.

Low virulent OUR T88/3 showed a 10-fold decrease in titre in the presence of IFN compared to virulent strains of ASFV. There are a number of differences between OUR T88/3 and related virulent viruses that could account for the differing sensitivity to IFN (Chapman et al., 2008, Portugal et al., 2015). Major differences include disruption of the EP153R and EP402R genes responsible for haemadsorption and variations in the number of copies of MGF110, MGF360 and MGF505. Of particular interest were the deletion of five members of MGF360 and two of MGF505 as these have been linked to the induction of IFN (Afonso et al., 2004). MGF360 and MGF505 members have no sequence identity with other cellular or viral proteins. The role of MGF360/505 genes in sensitivity of ASFV replication were explored further using recombinant Pr4 strains that lacked different combinations of these MGF genes. However, whilst Pr4Δ35, a strain lacking a similar subset of MGF genes to OUR T88/3 was susceptible to IFN, strains lacking only subsets of these genes were not, suggesting that no single gene is sufficient to enable ASFV to replicate optimally in the presence of IFNα. One interesting result was that in our hands Pr4Δ35 replicated as efficiently as wild type Pr4 in both alveolar (Fig. 2b) and blood derived macrophages (Supplemental Fig. 2b). Previously this deletion mutant has been shown to have a thousand-fold growth defect in blood derived macrophages (Burrage, et al. 2004). The only differences between our experiments and the published data is that we cultured macrophages with porcine serum, whereas Burrage et al. used L929 supernatant and foetal bovine serum, therefore there may have been differences in the maturation states of the macrophage cultures. Foetal calf serum has been linked to reduced virus growth in alveolar macrophages (Bustos, et al., 2002), but it seems unlikely that this would account for the difference we have observed. OUR T88/3 and recombinant Pr4Δ35, which were sensitive to IFNα, lack both MGF505-1R and MGF505-2R when compared to virulent strains. However, the other recombinant Pr4 viruses that were not inhibited by IFN contained one of either MGF505-1R or MGF505-2R. Therefore, it is possible that efficient ASFV replication in the presence of IFN requires either MGF505-1R or MGF505-2R, but the loss of both genes reduces ASFV replication in the presence of IFN. Reintroduction of individual members of MGF360 or MGF505 into the Pr4Δ35 or OUR T88/3 backbone would show which genes are required for replication in the presence of IFN.

Low virulent OUR T88/3 induced IFN *in vitro* in DCs, was sensitive to pre-treatment with IFNα, did not induce any detectable levels of IFN *in vivo* and can induce robust immunity to related virulent strains of ASFV (Boinas et al., 2004, King et al., 2011, Oura et al., 2005). It is possible that induction of IFN in target cells and or DCs in lymphoid tissues coupled with the sensitivity of OUR T88/3 to IFN may limit virus replication and hence dissemination of the virus and therefore the level of circulating IFN induced *in vivo*. Nevertheless, the ability of OUR T88/3 to induce IFN in DCs may be sufficient to activate DCs and natural killer cells as well influencing T-cell responses. Both CD8 cells and NK cells play an important role in protective immunity against ASFV (Leitao et al., 2001, Oura et al., 2005).

## 4 Methods

### 4.1 Cells

Porcine primary cells were derived from Large White outbred pigs typically 4 weeks old. Bone marrow cells were prepared from femur bones and were maintained in EBSS (Sigma) supplemented with 10% porcine serum (BioSera) and 100 IU/ml penicillin and 100 µg/ml streptomycin. Alveolar macrophages were prepared by lung lavage with Ca/Mg-free PBS and maintained in RPMI 1640 medium supplemented with 10% heat-inactivated porcine serum and 200 IU/ml penicillin and 200 µg/ml streptomycin. Vero and Madin–Darby bovine kidney (MDBK*t*2) cells were maintained in DMEM-HEPES supplemented with 10% heat-inactivated foetal calf serum and 100 IU/ml penicillin and 100 µg/ml streptomycin. 10 µg/ml Blasticidin was included with the MDBK*t*2 cells.

### 4.2 Antibodies

The following monoclonal antibodies were used for depletion studies and flow cytometry: anti-CD3 (PPT3, IgG1) (Yang and Parkhouse, 1996), anti-CD21 (CC51, IgG2b) (Saalmuller, 1996), anti-CD14 (CCG33, IgG1) (Sopp et al., 1996), anti-CD4 (PT90A, IgG2a; VMRD, USA), and anti-CD172 (74-22-15; IgG1) (Saalmuller, 1996). Monoclonals against bovine WC1 (CC39; IgG1, CC15; IgG2a) (Morrison and Davis, 1991) and bovine CD6 (CC38; IgG2b) (Letesson and Bensaid, 1991) were used as isotype controls.

### 4.3 Flow cytometry

1 μg of purified monoclonal antibody or 15 μl of tissue culture supernatant was labelled using 5 μl of Zenon antibody labelling kits (Life Technologies), using Alexa Fluor® 488 and R-Phycoerythrin. 3×10^5^ cells were incubated with Live/Dead® Fixable Near-IR Dead cell stain (Life Technologies), conjugated antibodies, and fixed with 1% PFA prior to acquisition on the LSR Fortessa (BD Biosciences). Analysis was carried out on FlowJo software where cells were gated on live, single cells with 99% viability observed.

### 4.4 Viruses

The viruses used in these experiments have been previously described, and include ASFV field strains: virulent Georgia 2007/1 (Rowlands et al., 2008), virulent Badajoz 1971 (BA71) (Enjuanes et al., 1976), virulent OUR T88/1, low virulent OUR T88/3 (Boinas et al., 2004), and virulent Pretoriuskop/96/4 (Pr4) (Kleiboeker et al., 1998). Recombinant Pr4 mutants strains Pr4Δ35, Pr4Δ3-C1, Pr4Δ3-C2, Pr4Δ5-1, Pr4Δ5-2 have been described previously (Burrage et al., 2004) and were a kind gift of Laslo Zsak and John Neilan (Plum Island Animal Disease Center). ASFV strains were grown in primary porcine bone marrow cells and mock inoculum were prepared from uninfected cells. Suid herpesvirus 1 (SuHV-1) was grown in Vero cells.

### 4.5 Animal experiments

All experiments were carried out under Home Office license PPL 70/7198 which was approved by the Ethical Review Committee of The Pirbright Institute. Three outbred Large White-Landrace pigs weighing approximately 20 kg were inoculated intramuscularly with 10^4^ HAD_50_ of virulent Georgia 2007/1 (animal numbers AP55 to AP57). Pigs inoculated intramuscularly with 10^4^ HAD_50_ of virulent OUR T88/1 (animal numbers AP19 to AP21) have been described previously (Abrams et al., 2013). Six NIH minipigs of swine leucocyte antigen (SLA) CC and DD haplotypes (SPF free) weighing between 18 and 44 kg were inoculated intramuscularly with 10^4^ TCID_50_ of low virulent OUR T88/3 (Animal numbers C924, C926, C927, D784, D787 and D792).

### 4.6 IFNα treatment

Porcine alveolar macrophages were pretreated for 24 h with 2000 IU/ml of recombinant porcine IFNα1 (PBL Interferon Source). Virus inoculum was diluted in serum free media and then added to cells at a multiplicity of infection (MOI) of 0.1. After 90 min at 37 °C with 5% CO_2,_ the inoculum was removed and cells were washed with Ca/Mg-free PBS three times. Infection was stopped after 48 h and the cells underwent one freeze/thaw cycle at −80 °C before virus titres were determined by end-point dilution in alveolar macrophages. Logarithmic virus titres were then calculated using the Spearman and Kärber method. Levels of virus in the blood of infected pigs were determined using quantitative PCR as described previously (King et al., 2011).

### 4.7 Detection of biologically active IFN

MDBK*t*2 cells, in which the chloramphenicol acetyltransferase (CAT) gene is under the control of the human MxA promoter (Fray et al., 2001) were used to determine the presence of biologically active IFN. A two-fold serial dilution of recombinant porcine IFNα1 (range 1000 IU/ml to 7.5 IU/ml) was tested in duplicate and used as a reference to determine the level of IFN present in serum derived from ASFV-infected pigs. A media only control was included. The expression of CAT was determined by ELISA (Roche).

### 4.8 Detection of IFNα and IFNβ

Solid sandwich ELISA detecting porcine IFNα has been described previously (Diaz de Arce et al., 1992). Briefly monoclonal anti-IFNα antibodies K9 (PBL interferon source) and F17 (PBL interferon source), were used as antigen capture and detecting antibodies, respectively. The K9 antibody was diluted to 5 μg/ml in carbonate/bicarbonate buffer and F17 antibody was labelled with biotin. TMB core substrate (AbDserotec, Bio-Rad) was added and developed for 10 min at room temperature. The reaction was terminated by the addition of 100 μl of 1 M H_2_SO_4_, and absorbance read at 492 nm. The level of IFNβ was determined using a porcine IFNβ ELISA kit (Cusabio).

### 4.9 Depletion of CD3^+^, CD14^+^ and CD21^+^ from PBMCs and infection with ASFV

PBMCs were purified from citrated blood derived from three Babraham in-bred Large-white pigs by centrifugation over histopaque gradients. 3×10^8^ PBMCs were incubated at 4 °C for 15 min in an antibody mixture containing anti-CD3, anti-CD21 and anti-CD14 in 5% FCS/PBS. After washing twice in Ca/Mg-free PBS, anti-mouse IgG1 and anti-mouse IgG2 beads (MACS Miltenyl Biotec) were added and incubated and washed as before. The cells were passed through LS columns (MACS Miltenyi Biotec) and the flow-through was collected. After centrifugation, the cells were resuspended in DMEM+HEPES supplemented with 10% pig serum, 100 µg/ml streptomycin and 100 U/ml penicillin to a density of 5×10^6^ cells/well. Cells were infected with ASFV strains OUR T88/1 and OUR T88/3 at an MOI of 1 or mock infected. CpG ODN 2216 (InvivoGen) at a final concentration of 3.2 µg/ml or 2 hemagglutination units of Sendai virus (Charles River Laboratories International) were added as positive controls. Biologically active IFN was determined from cell supernatants 24 hours post infection using the MxCAT assay (Fray et al., 2001). The mock inoculum and the two ASFV strains were also tested for their ability to induce CAT expression in the MDBKt2 cells in the absence of the negatively-selected PBMCs.

### 4.10 Statistical analysis

Statistical analysis was carried out using Minitab 16 statistical analysis programme, where a one-way ANOVA was carried out with Tukey׳s *post-hoc* test when a difference was observed.
