# Supplementary material for: Sensitivity of African swine fever virus to type I interferon is linked to genes within multigene families 360 and 505
Source: Virology. 2016 Jun;493:154–61. doi: 10.1016/j.virol.2016.03.019 (PMC4863678; doi:10.1016/j.virol.2016.03.019)
Supplement: Supplementary file 1 — Supplementary material [file mmc1.pdf]

Supplementary Material For: "Sensitivity of African swine fever virus to type I interferon is linked to genes within multigene families 360 and 505." Josephine P. Golding, Lynnette Goatley, Steve Goodbourn, Linda K. Dixon, Geraldine Taylor and Christopher L. Netherton

Supplementary Text

Serum samples from outbred pigs infected with the attenuated OUR T88/3 isolate of ASFV were tested for the presence of biological interferon (IFN) activity using the MDBKt2/CAT assay. The level of IFN in the serum of the five pigs did not alter appreciably during the course of the experiment (Supplementary Fig. 1). Coupled with the results from inbred pigs (Fig. 1f) these data show that attenuated OUR T88/3 does not induce systemic levels of IFN comparable to that seen in pigs infected with virulent ASFV (Fig. 1a, b).

Experiments in alveolar macrophages showed that replication of OUR T88/3 and Pr4Δ35 ASFV strains were inhibited by pre-treatment with IFN, but that virulent strains were not (Fig. 2a, b). These experiments were then repeated in blood derived macrophages to examine if this were true for macrophages derived from a different source. Macrophage cultures were treated for 24 hours with different concentrations of IFN and then infected with virulent OUR T88/1, attenuated OUR T88/3 or with Suid herpesvirus 1 (SuHV-1). Titres of OUR T88/3 and SuHV-1 were reduced by pretreatment by both 200 and 2000 IU/ml of IFN (Supplementary Fig. 2a). In a subsequent experiment, macrophages cultured from a different animal were pretreated for 24 hours with 2000 IU/ml of IFN and then infected with virulent OUR T88/1, virulent Pr4, virulent Malawi Lil 20/1, attenuated OUR T88/3 and recombinant Pr4Δ35, as well as with Suid herpesvirus 1 (SuHV-

1). Replication of the three virulent viruses were not reduced by IFN pretreatment, whereas OUR T88/3, Pr4Δ35 and SuHV-1 were significantly reduced (p = 0.016, 0.0036, 0.016 respectively). This shows that the ability of virulent ASFV to replicate in the presence of IFN is not dependent on the source of the macrophage cultures, likewise attenuated ASFV replication is inhibited by IFN in both alveolar and blood-derived macrophages. Note that the titre of Pr4Δ35 was lower than that of parental Pr4 in control cultures that were not treated with IFN, but this was not mathematically significant (p = 0.065). We did not see the 2.5 to 3 log difference in titre between Pr4 and Pr4Δ35 previously reported (Zsak *et al.*, 2001) in either macrophages derived from blood (Supplementary Fig. 2b) or lung lavage (Fig. 2b)

Macrophage cultures derived from two different pigs were infected with either OUR T88/1 or OUR T88/3 (Supplementary Fig. 3a), or Pr4 or Pr4Δ35 (Supplementary Fig. 3b). The supernatants were then tested for the presence of IFN using the MDBKt2/CAT bioassay 48 hours post infection. In the first experiment levels of IFN above background were detected in supernatants from macrophages infected with OUR T88/3, but not OUR T88/1. This is consistent with previous experiments showing that attenuated NHP/68 induce IFN, but virulent Lisbon 60 does not (Gil *et al.*, 2008). OUR T88/1, OUR T88/3, Lisbon 1960 and NHP/68 are all European genotype I ASFV isolates and the OUR T88/3 and NHP/68 genomes are 99.99% identical (Portugal *et al.*, 2015). Similarly Pr4 did not induce IFN, but Pr4Δ35 did which is consistent with a previous comparison of IFN induction between these two viruses (Afonso *et al.*, 2004). In combination with results from other groups this data suggests that virulent ASFV strains do not induce IFN in *in vitro*

macrophage cultures, whereas attenuated strains which lack members of MGF360 and MGF505 do.

Supplementary Methods

Viruses: The viruses used in these experiments have been previously described, and include ASFV field strains: virulent OUR T88/1, low virulent OUR T88/3 (Boinas et al., 2004), virulent Pretoriuskop/96/4 (Pr4) (Kleiboeker et al., 1998) and virulent Malawi Lil 20/1 (Haresnape et al., 1988). Recombinant Pr4 mutant strain Pr4Δ35 has been described previously (Burrage et al., 2004) and was a kind gift of Laslo Zsak and John Neilan (Plum Island Animal Disease Center). ASFV strains were grown in primary porcine bone marrow cells and mock inoculum were prepared from uninfected cells. Suid herpesvirus 1 (SuHV-1) was grown in Vero cells. Sendai Virus (SeV), Cantell Strain (ATCC VR-907 Parainfluenza 1) was purchased from Charles River, Wilmington MA, USA (#10100773).

Cells: Porcine primary cells were derived from Large White outbred pigs typically 4 weeks old and cultured at 37°C and 5% CO_2 in a humidified incubator.

Bone marrow cells were prepared from femur bones and were cultured from three days in EBSS (Sigma) supplemented with 10% porcine serum (BioSera) and 100 IU/ml penicillin and 100 µg/ml streptomycin.

Blood derived macrophages were prepared from whole blood by a modification of a protocol described previously (McCullough et al., 1999). Erythrocytes were sedimented by incubating 6 % dextran (Sigma) in 0.85 % saline solution with an equal volume of whole blood at 37 °C for 30

minutes. The upper fraction containing peripheral blood leukocytes was removed and washed twice with Ca/Mg free PBS (Sigma). Any remaining erythrocytes were lysed by the addition of 5 ml of ice cold sterile distilled water followed by the addition of 5 ml of ice cold serum-free DMEM medium (Sigma), and were left on ice for 5 minutes. Cells were collected by centrifugation (450 g, 5 min, 4°C) and then resuspended in DMEM + HEPES (Life Tech) supplemented with 30 % (v/v) pig serum, 200 µg/ml streptomycin and 200 U/ml penicillin. Adherent cells were selected by 2 hours incubation on plastic cell culture plates, non-adherent cells were removed and fresh media added. 3 days later the media was replace with Earles media supplemented with 30 % (v/v) pig serum, 200 µg/ml streptomycin and 200 U/ml penicillin, and the cells were cultured for a further day.

Madin-Darby bovine kidney cells encoding the chloramphenicol acetyltransferase (CAT) gene under the control of the human MxA promoter (MDBK*t*2) have been described previously (Fray et al., 2001) and were maintained in DMEM-HEPES supplemented with 10% heat-inactivated foetal calf serum and 100 IU/ml penicillin, 100 µg/ml streptomycin and 10 µg/ml Blasticidin.

Animal experiment: All experiments were carried out under Home Office license PPL 70/7198 which was approved by the Ethical Review Committee of The Pirbright Institute. Five outbred Large White-Landrace pigs weighing approximately 20 kg were inoculated intramuscularly with 10^4 $TCID_{50}$ of attenuated OUR T88/3 (animal numbers AS58 to AS62).

IFNα treatment: Macrophages were pretreated for 24 hours with recombinant porcine IFNα1 (PBL Interferon Source, UK). Virus inoculum was diluted in serum free media and then added to

cells at a multiplicity of infection (MOI) of 0.1. After 90 minutes at 37°C with 5% CO_2, the inoculum was removed and cells were washed with Ca/Mg-free PBS three times. Infection was stopped after 48 hours and the cells underwent one freeze/thaw cycle at -80°C before virus titres were determined by end-point dilution in bone marrow macrophages. Logarithmic virus titres were then calculated using the Spearman and Kärber method.

Detection of biologically active IFN: MDBK*t*2 cells were used to determine the presence of biologically active IFN. A two-fold serial dilution of recombinant porcine IFNα1 (range 1000 IU/ml to 7.5 IU/ml) was tested in duplicate and used as a reference to determine the level of IFN present in serum derived from ASFV-infected pigs. A media only control was included. The expression of CAT was determined by ELISA (Roche).

References

Afonso C.L., Piccone M.E., Zaffuto K.M., Neilan J., Kutish G.F., Lu Z., Balinsky C.A., Gibb T.R., Bean T.J., Zsak L. & Rock D.L. (2004). African swine fever virus multigene family 360 and 530 genes affect host interferon response. *J. Virol* **78**, 1858-1864.

Fray M. D., Mann G. E. & Charleston, B. (2001). Validation of an Mx/CAT reporter gene assay for the quantification of bovine type-I interferon. *J. Imm. Met.* **249**, 235-244.

Gil S., Sepúlveda N., Albina E., Leitão A. & Martins C., (2008). The low-virulent African swine fever virus (ASFV/NH/P68) induces enhanced expression and production of relevant regulatory

cytokines (IFNalpha, TNFalpha and IL12p40) on porcine macrophages in comparison to the highly virulent ASFV/L60. *Arch. Virol.* **153**, 1845-1854.

Haresnape J. M., Wilkinson P. J. & Mellor P. S. (1988). Isolation of African swine fever virus from ticks of the Ornithodoros moubata complex (Ixodoidea: Argasidae) collected within the African swine fever enzootic area of Malawi. *Epidemiol Infect* **101**, 173-185.

McCullough K. C., Basta S., Knotig S., Gerber H., Schaffner R., Kim Y. B., Saalmuller A. & Summerfield A. (1999). Intermediate stages in monocyte macrophage differentiation modulate phenotype and susceptibility to virus infection. *Immunol.* **98**, 203-212.

Portugal R., Coelho J., Höper D., Little N.S., Smithson C., Upton C., Martins C., Leitão A. & Keil G.M. (2015). Related strains of African swine fever virus with different virulence: genome comparison and analysis. *J. Gen. Virol.* **96** 408-419.

Zsak L., Lu Z., Burrage T.G., Neilan J.G., Kutish G.F., Moore D.M. & Rock D.L. (2001). African swine fever virus multigene family 360 and 530 genes are novel macrophage host range determinants. *J. Virol* **75**, 3066-3076.

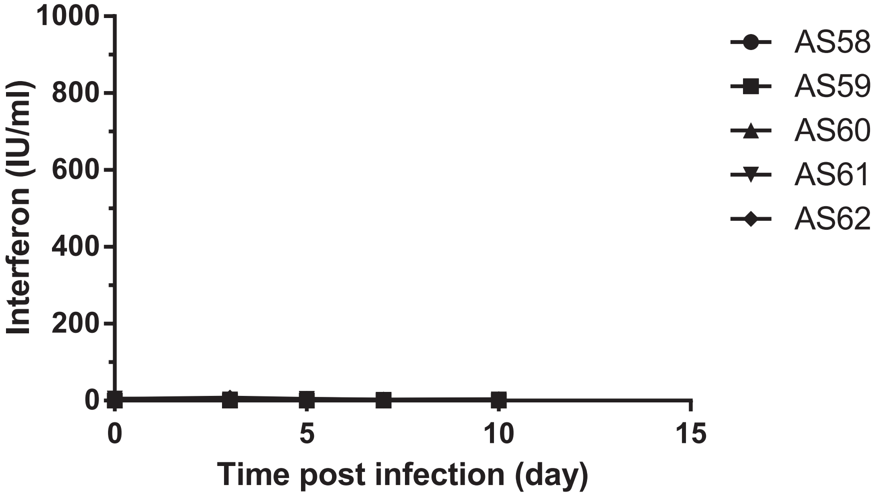

Supplementary Figure 1: IFN in the serum of pigs infected with ASFV OUR T88/3 in outbred pigs. Serum was collected from Large White-Landrace pigs inoculated intramuscularly with 10^4 HAD50 of virulent OUR T88/3. Biologically active IFN was determined using the MDBkt2/CAT bioassay. Results are expressed as the mean biological activity of duplicate serum samples. AS58, AS59 etc. correspond to individual animal numbers

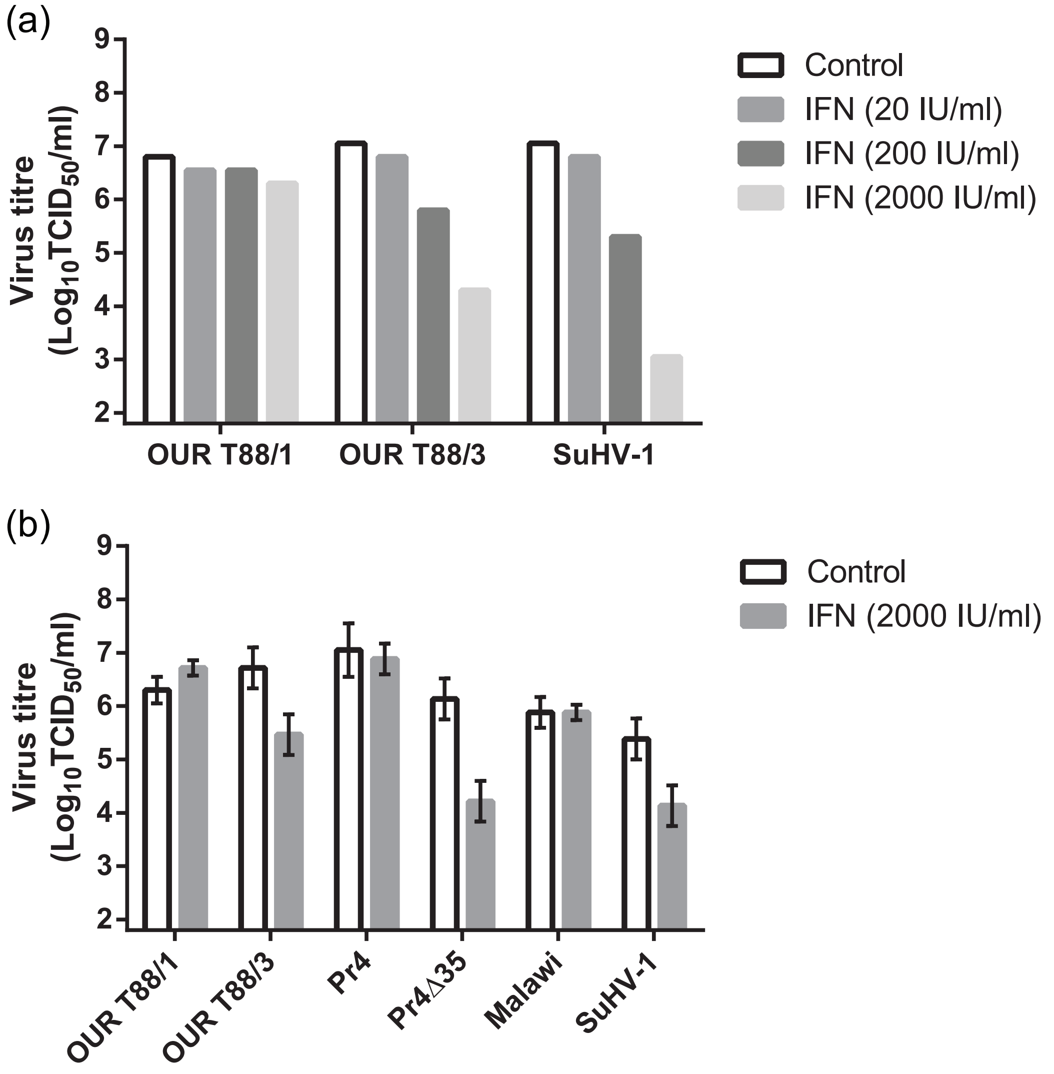

Supplemental figure 2: IFN inibits replication of attenuated ASF in blood derived macrophage cultures. Macrophages cultured from two different pigs (a, b) and then treated for 24 hours with the indicated amount of recombinant porcine IFNα (grey bars) or left untreated (white bars). Cells were then infected with the indicated ASFV strains or suid herpesvirus 1 (SuHV-1) at an MOI of 0.1 and virus yields determined 48 hours later. Virus titres are either shown as the result of single well per condition (a) or as the mean of three independent wells ± standard deviation (b).

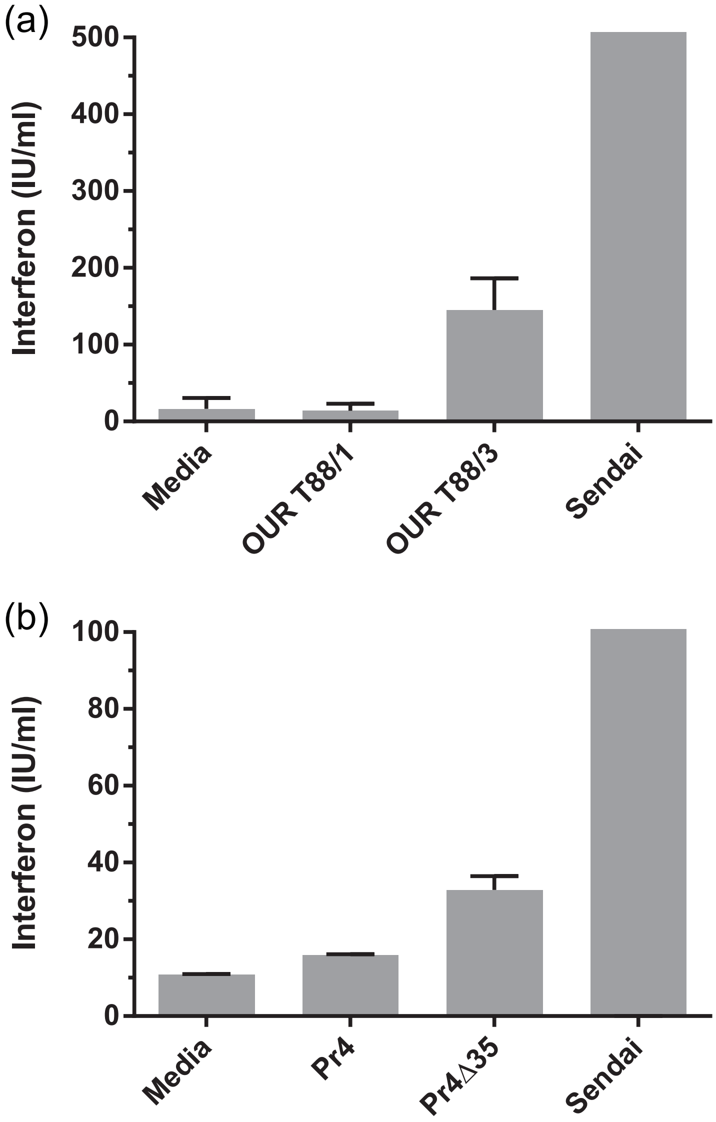

Supplementary Figure 3: Induction of IFN in porcine macrophage cultures infected with virulent and attenuated ASFV strains. Bone marrow macrophage cultures derived from pig 1 were infected with virulent OUR T88/1 or avirulent OUR T88/3 (a) and cells derived from pig 2 were infected with virulent Pr4 or recombinant Pr4Δ35 (b). Sendai virus was used a positive control for the induction of IFN and cells in media only were used as a negative control. A MOI of 1 was used for each ASFV strain. Supernatants were collected 48 hours post infection and tested for the presence of IFN by MDB-Kt2/CAT bioassay. Results are expressed as the mean biological activity of duplicate samples ± standard deviation, the bars representing data from the Sendai samples have been cropped at 500 or 100 IU/ml for clarity.
